# Host, reproductive, and lifestyle factors in relation to quantitative histologic metrics of the normal breast

**DOI:** 10.1186/s13058-023-01692-7

**Published:** 2023-08-15

**Authors:** Mustapha Abubakar, Alyssa Klein, Shaoqi Fan, Scott Lawrence, Karun Mutreja, Jill E. Henry, Ruth M. Pfeiffer, Maire A. Duggan, Gretchen L. Gierach

**Affiliations:** 1 Division of Cancer Epidemiology and Genetics, National Cancer Institute, National Institutes of Health, 9609 Medical Center Drive, Shady Grove, Bethesda, MD 20850 USA; 2 Molecular and Digital Pathology Laboratory, Cancer Genomics Research Laboratory, Leidos Biomedical Research, Inc., Frederick, MD 21702 USA; 3 Biospecimen Collection and Banking Core, Susan G. Komen Tissue Bank at the IU Simon Comprehensive Cancer Center, Indianapolis, IN USA; 4 Department of Pathology and Laboratory Medicine, University of Calgary, Calgary, AB T2N2Y9 Canada

**Keywords:** Breast cancer, Etiology, Risk factors, Parity, Body mass index, Race, Ethnicity, Family history, Breast tissue composition, Normal breast, Epithelium, Stroma, Epithelium-to-stroma proportion

## Abstract

**Background:**

Emerging data indicate that variations in quantitative epithelial and stromal tissue composition and their relative abundance in benign breast biopsies independently impact risk of future invasive breast cancer. To gain further insights into breast cancer etiopathogenesis, we investigated associations between epidemiological factors and quantitative tissue composition metrics of the normal breast.

**Methods:**

The study participants were 4108 healthy women ages 18–75 years who voluntarily donated breast tissue to the US-based Susan G. Komen Tissue Bank (KTB; 2008–2019). Using high-accuracy machine learning algorithms, we quantified the percentage of epithelial, stromal, adipose, and fibroglandular tissue, as well as the proportion of fibroglandular tissue that is epithelium relative to stroma (i.e., epithelium-to-stroma proportion, ESP) on digitized hematoxylin and eosin (H&E)-stained normal breast biopsy specimens. Data on epidemiological factors were obtained from participants using a detailed questionnaire administered at the time of tissue donation. Associations between epidemiological factors and square root transformed tissue metrics were investigated using multivariable linear regression models.

**Results:**

With increasing age, the amount of stromal, epithelial, and fibroglandular tissue declined and adipose tissue increased, while that of ESP demonstrated a bimodal pattern. Several epidemiological factors were associated with individual tissue composition metrics, impacting ESP as a result. Compared with premenopausal women, postmenopausal women had lower ESP [*β* (95% Confidence Interval (CI)) = −0.28 (− 0.43, − 0.13); *P* < 0.001] with ESP peaks at 30–40 years and 60–70 years among pre- and postmenopausal women, respectively. Pregnancy [*β* (95%CI) _vs nulligravid_ = 0.19 (0.08, 0.30); *P* < 0.001] and increasing number of live births (*P*_-trend_ < 0.001) were positively associated with ESP, while breastfeeding was inversely associated with ESP [*β* (95%CI) _vs no breastfeeding_ = −0.15 (− 0.29, − 0.01); *P* = 0.036]. A positive family history of breast cancer (FHBC) [*β* (95%CI) _vs no FHBC_ = 0.14 (0.02–0.26); *P* = 0.02], being overweight or obese [*β* (95%CI) _vs normal weight_ = 0.18 (0.06–0.30); *P* = 0.004 and 0.32 (0.21–0.44); *P* < 0.001, respectively], and Black race [*β* (95%CI) _vs White_ = 0.12 (− 0.005, 0.25); *P* = 0.06] were positively associated with ESP.

**Conclusion:**

Our findings revealed that cumulative exposure to etiological factors over the lifespan impacts normal breast tissue composition metrics, individually or jointly, to alter their dynamic equilibrium, with potential implications for breast cancer susceptibility and tumor etiologic heterogeneity.

**Supplementary Information:**

The online version contains supplementary material available at 10.1186/s13058-023-01692-7.

## Introduction

Risk factors for breast cancer development are thought to promote carcinogenesis by inducing proliferative epithelial changes, but emerging data suggest that stromal and adipose tissue components of the breast may also play crucial roles in early stages of breast carcinogenesis [1–3]. Results from a previous study by Troester and colleagues, for example, found that associations between some risk factors and involution of the breast epithelium were modified by the mammary stroma [4]. Further, adipose tissue features, such as crown-like structures, that are reflective of increased levels of proinflammatory mediators, aromatase expression, and possibly elevated breast cancer risk, have been found to be more prevalent among obese than normal weight postmenopausal women [5].

In support of a stromal role in breast cancer etiopathogenesis, a recent study by our group found a context-dependent role of the stroma to either prevent or promote breast cancer development in women with benign breast disease (BBD) [6]. Among BBD patients with non-proliferative disease, we observed increasing stromal proportion to be strongly protective against breast cancer development, whereas among those with proliferative disease increasing stroma was associated with increasing breast cancer risk. We also found the relative abundance of epithelium to stroma on BBD biopsies, i.e., the epithelium-to-stroma proportion (ESP), to be strongly associated with risk of future invasive breast cancer, independently of BBD histological classification [6]. In another study of BBD patients from the Nurses’ Health Study (NHS), Vellal and colleagues found a similar metric on BBD biopsies, i.e., the epithelium-to-stroma ratio (ESR), to be independently associated with elevated risk of future invasive breast cancer [7]. In both studies, the association between ESP/ESR and breast cancer risk was stronger among women with non-proliferative than proliferative BBD.

Morphologically, non-proliferative diseases more closely resemble the normal adult female breast than proliferative diseases [8]. Accordingly, results from previous studies may be indicative of the role of disruptive changes in the epithelial and stromal equilibrium in the pathogenesis of breast cancer [6, 7]. Most previous studies that have examined the association between epidemiological factors and tissue composition metrics have relied on measures of involuting epithelial structures called terminal duct lobular units (TDLUs) and/or were largely conducted within BBD populations [9–16]. Both have limitations. TDLUs do not capture information on tissue composition metrics beyond epithelial changes, and results from BBD populations may be limited by the impact of the underlying BBD pathology on breast tissue composition. For example, results from our previous BBD study were in support of associations between individual breast cancer risk factors and quantitative tissue composition metrics, including ESP, but the underlying BBD lesion was the strongest predictor of variations in breast tissue composition [6].

The main aim of this study was, therefore, to investigate the associations between several breast cancer risk factors and quantitative tissue composition metrics in normal breast biopsies, individually (epithelium, stroma, and adipose tissue) and in combination as fibroglandular tissue (epithelium plus stroma) and ESP (the proportion of fibroglandular tissue that is epithelium, relative to stroma), among women participating in the Susan G. Komen Tissue Bank.

## Methods and materials

### Study population

Participants in this study were women without a personal history of breast cancer who voluntarily donated breast tissues to the Susan G. Komen Tissue Bank (KTB). Details of the KTB project (http://komentissuebank.iu.edu/) have been described elsewhere [17–19]. In brief, the KTB is a continuously growing biorepository that collects, stores, and annotates histologically normal breast tissue donated by volunteers. Participants were generally women ≥ 18 years of age at donation with no breast implants and not receiving strong blood thinners or radiation to the chest. About 5382 tissue donations from 4906 women were recorded in the KTB by 2019. For women with multiple donations (*n* = 476), we used the earliest donation corresponding to the time when questionnaires were administered. Women without hematoxylin and eosin (H&E)-stained images (*n* = 526) were excluded from the analytical population. In addition, we excluded women above 75 years of age (*n* = 51) and those without data on age (*n* = 3), those who were pregnant and/or breastfeeding at the time of tissue collection (*n* = 59), previously had breast cancer (*n* = 149), and those without data on menopausal status (*n* = 10). The final analytical population comprised 4108 women who donated tissues between 2008 and 2019 and for whom we could retrieve the corresponding digitized H&E-stained sections (Additional file 1: Fig. S1). At the time of donation/enrollment, the participants provided written informed consent and were enrolled under a protocol approved by the Indiana University Institutional Review Board and the National Institutes of Health Office of Human Subjects Research (NIH OHSR #4508).

### Exposure assessment

The methodology for exposure assessment within KTB has been described elsewhere [13, 18]. In general, detailed information on sociodemographic, medical, reproductive, menstrual, and lifestyle factors, as well as information on gynecologic surgeries and mammographic screening, were collected by means of self-administered questionnaires. Relevant exposures for this analysis included age (years; < 30, 30–39, 40–49, 50–59, 60–75) at the time of tissue donation, race/ethnicity (Black, White, Asian/Other), age at menarche (years; categorized as ≤ 12, 13, ≥ 14), parity (gravid vs nulligravid), number of live births (0, 1, 2, ≥ 3), age at first full term birth (years; < 25, 25–29, ≥ 30), breastfeeding (ever vs never; duration), body mass index (BMI; < 25, 25–29, ≥ 30 kg/m^2^;), hormonal birth control use (yes vs no), menopausal status (post- vs. pre-menopausal), bilateral oophorectomy (yes vs none), menopausal hormone therapy (MHT) use (never, former, current) and MHT type, smoking status (never, former, current), alcohol intake, and family history of breast cancer (FHBC) in a first degree relative (present vs absent).

### Breast tissue collection

Up to four tissue cores were biopsied from the upper outer quadrant of the right or left breast using a standard 9-gauge (since 2010) or 10-gauge (prior to 2010) needle and one core was fixed in 10% buffered formalin [13, 18]. The formalin-fixed and paraffin-embedded (FFPE) tissue blocks that were prepared from that core were sectioned and stained using H&E staining according to standard laboratory procedures [13, 18]. Archival, digitized, H&E-stained sections were shared with the Molecular and Digital Pathology Laboratory (MDPL) of the Division of Cancer Epidemiology and Genetics (DCEG) at the National Cancer Institute (NCI), USA, for downstream tissue composition analysis (see below).

### Machine learning characterization of tissue composition metrics

**Fig. 1:**
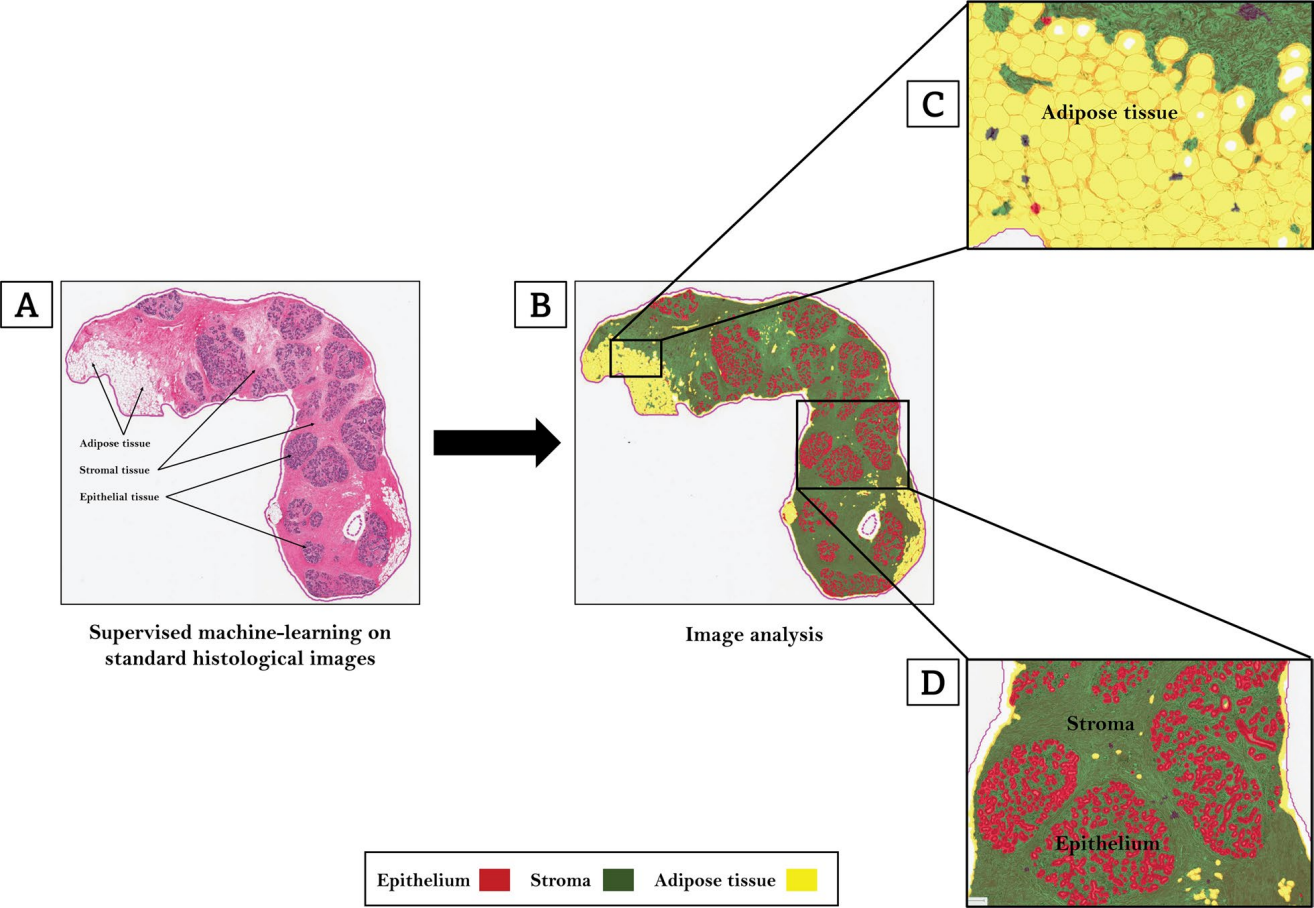
Machine learning analysis of quantitative tissue composition metrics. Digitized hematoxylin and eosin-stained slides were used to optimize machine learning-based tissue classification scripts. A custom-built, random forest, tissue classifier algorithm (Indica Labs, Albuquerque, NM) was trained by two pathologists to develop an optimized, 85-datapoint, tissue classifier script. By annotating regions on randomly selected representative H&E-images comprised of epithelium, stroma, and adipose tissue (**A**), the random forest algorithm was trained to identify, segment, and quantify areas (in mm.^2^) on each slide comprised of epithelium (42-datapoints), stroma (37-datapoints), and adipose tissue (6-datapoints) as shown on (**B**) (Red: epithelium; Green: stroma; Yellow: adipose tissue). **C** and **D** show high-power views of the machine’s capacity to identify regions on the slide comprised of adipose tissue (**C**) as well as epithelium and stroma (**D**)

Digitized H&E-stained slides were archived using the Halo Link digital image repository (Indica Labs, Albuquerque, NM) at the US National Cancer Institute (NCI). Image analysis was performed using the Halo Client computational pathology software (Indica Labs, Albuquerque, NM). A custom-built, random forest, tissue classifier algorithm was trained by two pathologists (MA and MAD) to develop an optimized, 85-datapoint, tissue classifier script. By annotating regions on randomly selected representative images comprised of epithelium, stroma, and adipose tissue, the random forest algorithm was trained to identify, segment, and quantify areas (in mm^2^) on each slide comprised of epithelium (42-datapoints), stroma (37-datapoints), and adipose tissue (6-datapoints) as shown on Fig. 1 (Red: epithelium; Green: stroma; Yellow: adipose tissue). In previous reproducibility analyses [6], we demonstrated excellent concordance (Spearman’s correlation coefficients ≥ 0.95) between scripts that were independently trained by two pathologists to identify and segment all three tissue types. Training and centralized image analysis were performed masked to all patient characteristics. Percent epithelium, stroma, and adipose tissue were calculated by dividing the absolute value of each histologic metric (in mm^2^) by the total tissue area (i.e., epithelium + stroma + adipose tissue, mm^2^) on each slide and multiplying by 100. Percent fibroglandular tissue area was calculated by adding epithelial and stromal area on the slides, dividing by total tissue area, and multiplying by 100. Percent ESP was calculated by dividing the epithelial area by total fibroglandular tissue area and multiplying by 100 as we previously described [6].

### Statistical analysis

Kruskal–Wallis tests were used to test differences in tissue composition metrics by participant characteristics. The associations of host (age, race/ethnicity, FHBC, menopause), reproductive (age at menarche, pregnant (gravid vs nulligravid), number of live births, age at first full-term birth (AFFB), breastfeeding), and lifestyle (smoking, alcohol intake, BMI, hormonal birth control, MHT use) factors with tissue composition metrics (epithelium, stroma, adipose tissue, fibroglandular tissue, and ESP) were assessed in linear regression models. All tissue composition metrics were square root transformed to better approximate the normal distributions for the linear regression model. Partially adjusted models included age and tissue area and fully adjusted models included all of the variables under consideration. Associations of breastfeeding, number of live births, and AFFB with tissue composition metrics were assessed in models restricted to previously pregnant women. Analyses were performed overall and stratified by menopausal status. In the overall model, bilateral oophorectomy and uterine ablation were included separately to examine their effects on tissue composition metrics. In stratified analyses, individuals who had a bilateral oophorectomy, irrespective of age, or uterine ablation after the age of 55 years, were considered postmenopausal. Locally weighted scatter plot smoothing (Lowess) functions were used to plot the residuals from multivariable linear regression models for each tissue composition metric as a function of age. Lowess plots were constructed overall, and separately for pre- and postmenopausal women, parous and nulliparous women, normal and overweight/obese women, and for Black and White women. For racial/ethnicity comparisons, plots were restricted to comparisons between Black and White women due to the very small numbers of other individual racial and ethnic groups. To explore whether BMI impacted parity and race-related curves, we conducted sensitivity analyses by stratifying Lowess plots for parity and race by BMI categories (i.e., normal versus overweight or obese). In sensitivity analyses, we also assessed whether parity impacted the race-related curves by creating separate plots for nulliparous and parous women. The majority of the risk factors were complete for participants. For those with missing values (Additional file 5: Table S1), however, these were addressed by the inclusion of missing values indicators in the models. In sensitivity analyses, we compared with multiple imputation and found the results to be similar (Additional file 5: Table S2). Further, we removed AFFB, which had the largest number of missing values (48.9%) from our model and compared models with and without AFFB (Additional file 5: Table S3). Although the results were similar, the model containing AAFB explained more variability in ESP than the model without AFFB (0.057 vs 0.045, respectively). Accordingly, AAFB was retained in models. All analyses were performed using R version 4.2 and all *p* values were two sided. Lowess plots were created using Stata statistical software version 16.1.

## Results

### Descriptive characteristics of analytical population

The characteristics of study participants are shown in Table 1. On average, participants were 43.8 years at the time of tissue donation (range = 18–75 years). Of the 4108 participants, 2696 (65.6%) were premenopausal while 1412 (34.4%) were postmenopausal. The majority (72%) of the participants were Non-Hispanic White, while ~ 18% were Black or African American and 9% were Asian or belonged to other ethnic groups (including Native Hawaiian/Pacific Islander, Alaskan native, Filipino, Japanese, Mixed race, and others). Most of the participants had a college degree (32%) or graduate/professional degree (25%). Two-thirds of the participants were either overweight or obese (BMI > 25 kg/m^2^).

**Table 1:** Characteristics of women volunteers who donated normal breast tissue to the US-based Susan G. Komen Tissue Bank that were included in the current study (*N* = 4108)

Characteristic	Overall, *N* (%)
*Age, years*	
< 30	819 (20.0)
30–39	822 (20.0)
40–49	909 (22.1)
50–59	913 (22.2)
60–75	645 (15.7)
*Race/ethnicity*	
Non-Hispanic White	2953 (71.9)
Non-Hispanic Black/African American	756 (18.4)
Asian/other	381 (9.3)
*Education*	
High school/GED or less	687 (16.7)
Vocation/tech school or associates degree	694 (16.9)
College degree	1331 (32.4)
Graduate/professional degree	1037 (25.2)
Other	338 (8.2)
*Smoking*	
Never	2780 (67.7)
Former	842 (20.5)
Current	137 (3.3)
*Currently drink alcohol*	
No	1372 (33.4)
Yes	2699 (65.7)
*Alcoholic drinks per week*	
< 1	284 (6.9)
1–6	1976 (48.1)
≥ 7	252 (6.1)
*Body mass index, kg/m*^*2*^	
< 25	1294 (31.5)
25–29	1102 (26.8)
≥ 30	1708 (41.6)
*Age at menarche, years*	
≤ 12	2180 (53.1)
13	1098 (26.7)
≥ 14	826 (20.1)
*Current hormonal birth control use*^*a*^	
No	2255 (83.6)
Yes	441 (16.4)
*Pregnancy*	
Nulligravid	1258 (30.6)
Gravid	2849 (69.4)
*Number of live births*	
0	197 (6.9)
1	582 (20.4)
2	1184 (41.6)
≥ 3	719 (25.2)
*Age at first full-term birth, years*	
< 25	645 (22.6)
25–29	428 (15.0)
≥ 30	356 (12.5)
*Breastfeeding*	
Never	810 (28.4)
Ever	1818 (63.8)
*Duration of breastfeeding, months*	
Never	810 (28.4)
< 12	971 (34.1)
≥ 12– < 24	484 (17.0)
≥ 24	359 (12.6)
*Bilateral oophorectomy*^*b*^	
No	752 (53.2)
Yes	295 (20.9)
*Menopausal status*	
Premenopausal	2696 (65.6)
Postmenopausal	1412 (34.4)
*Menopausal hormone therapy use*^*c*^	
Nonuser	779 (55.2)
Current user	217 (15.4)
Former user	115 (8.1)
*Type of current menopausal hormone therapy*	
Estrogen only	79 (36.4)
Progesterone only	0 (0.0)
Combined estrogen and progesterone	87 (40.1)
*Type of former menopausal hormone therapy*	
Estrogen only	54 (46.9)
Progesterone only	0 (0.0)
Combined estrogen and progesterone	38 (33.0)
*Family history of breast cancer*	
Negative	3006 (73.2)
Positive	8.7 (20.4)

Asian/Other category comprised women who self-identified as Asian, Chinese, Filipino, Japanese, “Other” Asian, Asian/Native Hawaiian or Pacific Islander, etc. Pregnant: Gravid = women who had previously been pregnant, irrespective of the outcome; nulligravid = women who had never been pregnant. Number of Live Births (0 = previous pregnancies did not result in a live birth). Percentages shown might not total to 100 due to missingness

^a^Current hormonal birth control use among pre-menopausal women. ^b^Frequency of bilateral oophorectomy among postmenopausal women. ^c^Frequency of menopausal hormone therapy use among postmenopausal women

### Associations of age and menopausal status with breast tissue composition metrics

The distributions of all the tissue composition metrics varied statistically significantly by age and menopausal status (Table 2). With the exception of adipose tissue, which was higher among older than younger and among postmenopausal- than premenopausal women, the distributions of all other tissue composition metrics were higher among younger than older women and among premenopausal than postmenopausal women. In multivariable linear regression models (Table 3), increasing age remained statistically significantly associated with decreasing epithelium, stroma, and fibroglandular tissue and with increasing adipose tissue, but not with ESP. On the other hand, compared with premenopausal women, postmenopausal women had significantly lower epithelium and ESP (Table 3).

**Table 2:** Distributions of quantitative tissue composition metrics according to characteristics of women volunteers who donated normal breast tissue to the US-based Susan G. Komen Tissue Bank that were included in the current study (*N* = 4108)

Characteristic	Epithelium (%)		Stroma (%)		Adipose tissue (%)		Fibroglandular tissue (%)		Epithelium-to-stroma proportion (%)	
	Mean	Median (range)	Mean	Median (range)	Mean	Median (range)	Mean	Median (range)	Mean	Median (range)
Overall	2.1	0.89 (0.0–70.4)	24.6	17.2 (0.0–95.7)	73.3	81.0 (0.0–100.0)	26.7	18.9 (0.0–97.2)	9.2	5.9 (0.0–100.0)
*Age, years*										
< 30	3.0	1.46 (0.0–25.1)	35.3	29.0 (0.0–93.5)	61.8	68.0 (5.1–100.0)	38.3	32.0 (0.0–94.9)	9.0	5.6 (0.0–85.2)
30–39	2.8	1.31 (0.0–53.4)	25.9	18.7 (0.0–92.2)	71.3	79.1 (6.1–100.0)	28.7	20.9 (0.0–93.9)	10.1	7.7 (0.0–98.7)
40–49	2.2	1.02 (0.0–70.4)	22.4	16.3 (0.0–91.8)	75.4	82.3 (7.3–100.0)	24.6	17.7 (0.0–92.7)	9.8	6.6 (0.0–100.0)
50–59	1.3	0.65 (0.0–18.4)	21.0	14.7 (0.0–91.3)	77.7	84.2 (6.4–100.0)	22.3	15.8 (0.0–93.6)	8.3	5.2 (0.0–100.0)
60–75	1.0	0.49 (0.0–13.0)	17.9	11.0 (0.0–95.8)	81.1	88.4 (2.8–100.0)	18.9	11.6 (0.0–97.2)	9.0	4.7 (0.0–93.8)
*P* value		** < 0.0001**		** < 0.0001**		** < 0.0001**		** < 0.0001**		** < 0.0001**
*Race/ethnicity*										
Non-Hispanic Caucasian	2.0	0.90 (0.0–53.4)	25.3	18.0 (0.0–95.8)	72.7	80.5 (2.8–100.0)	27.3	19.5 (0.0–97.2)	8.9	5.7 (0.0–100.0)
Black/African American	2.2	0.89 (0.0–70.4)	21.5	15.1 (0.0–88.9)	76.3	83.3 (6.1–100.0)	23.7	16.7 (0.0–93.9)	9.9	6.7 (0.0–92.2)
Asian/other	2.2	0.89 (0.0–25.1)	25.8	17.6 (0.0–91.8)	72.0	81.3 (6.1–100.0)	28.0	18.7 (0.0–93.9)	10.4	6.5 (0.0–92.9)
*P* value		0.98		**0.02**		0.08		0.08		**0.01**
*Education*										
High school/GED or less	2.4	0.96 (0.0–70.4)	29.0	20.5 (0.0–93.3)	68.6	77.5 (5.1–100.0)	31.4	22.5 (0.0–94.9)	9.6	5.7 (0.0–100.0)
Vocation/tech school or associates degree	1.9	0.84 (0.0–21.3)	21.2	14.9 (0.0–95.8)	76.9	83.7 (2.8–100.0)	23.1	16.3 (0.0–97.2)	9.2	6.6 (0.0–100.0)
College degree	2.1	0.94 (0.0–26.6)	35.6	18.6 (0.0–92.7)	72.3	79.9 (5.7–100.0)	27.7	20.1 (0.0–94.3)	9.0	5.8 (0.0–93.7)
Graduate/professional degree	2.1	0.87 (0.0–53.4)	23.2	16.1 (0.0–93.1)	74.7	82.8 (6.4–100.0)	25.3	17.2 (0.0–93.6)	9.6	6.0 (0.0–98.7)
Other	1.9	0.85 (0.0–17.6)	24.0	17.3 (0.0–93.5)	74.1	80.8 (5.6–100.0)	25.9	19.2 (0.0–94.4)	8.5	5.7 (0.0–87.1)
*P* value		0.58		** < 0.0001**		** < 0.0001**		** < 0.0001**		0.34
*Smoking*										
Never	2.2	0.95 (0.0–53.4)	25.1	18.1 (0.0–95.8)	72.7	80.4 (2.8–100.0)	27.3	19.6 (0.0–97.2)	9.1	6.0 (0.0–100.0)
Former	1.6	0.72 (0.0–70.4)	22.9	15.7 (0.0–92.1)	75.5	83.3 (6.3–100.0)	24.5	16.7 (0.0–93.7)	9.0	5.3 (0.0–100.0)
Current	1.9	0.96 (0.0–9.76)	27.7	22.1 (0.0–92.5)	70.4	77.0 (5.7–100.0)	29.6	23.0 (0.0–94.8)	10.0	6.1 (0.0–93.7)
*P* value		**0.008**		0.13		**0.02**		**0.02**		**0.03**
*Currently drink alcohol*										
No	2.0	0.86 (0.0–70.4)	23.1	15.3 (0.0–95.8)	74.8	83.2 (2.8–100.0)	25.2	16.8 (0.0–97.2)	9.6	6.3 (0.0–100.0)
Yes	2.1	0.92 (0.0–53.4)	25.4	18.4 (0.0–93.1)	72.5	80.2 (5.1–100.0)	27.5	19.8 (0.0–94.9)	9.0	5.8 (0.0–100.0)
*P* value		0.73		**0.02**		0.69		**0.03**		0.06
*Average number of alcoholic drinks per week*										
< 1	2.0	0.84 (0.0–23.3)	22.9	13.8 (0.0–84.6)	75.1	84.1 (10.5–100.0)	24.9	15.9 (0.0–89.5)	10.9	7.0 (0.2–93.7)
1–6	2.2	0.91 (0.0–53.4)	25.6	18.8 (0.0–93.1)	72.2	79.1 (5.1–100.0)	27.8	20.3 (0.0–95.9)	8.8	5.6 (0.0–100.0)
≥ 7	1.7	0.92 (0.0–15.4)	26.8	18.7 (0.0–91.9)	71.5	69.9 (6.9–100.0)	28.5	20.1 (0.0–93.0)	7.2	4.7 (0.0–62.4)
*P* value		0.69		**0.003**		**0.006**		**0.006**		** < 0.0001**
*Body mass index*										
< 25	2.6	1.25 (0.0–53.4)	32.4	26.1 (0.0–95.8)	65.0	71.9 (2.8–100.0)	35.0	28.1 (0.0–97.2)	8.0	5.1 (0.0–98.7)
25–29	1.9	0.79 (0.0–36.2)	23.6	15.8 (0.0–93.5)	74.5	82.4 (5.6–100.0)	25.5	17.6 (0.0–94.4)	9.2	5.9 (0.0–100.0)
≥ 30	1.8	0.74 (0.0–70.4)	19.5	12.9 (0.0–92.4)	78.7	85.6 (6.1–100.0)	21.3	14.4 (0.0–93.9)	1.0	6.9 (0.0–97.5)
*P* value		** < 0.0001**		** < 0.0001**		** < 0.0001**		** < 0.0001**		** < 0.0001**
*Age at menarche, years*										
≤ 12	2.1	0.89 (0.0–70.4)	23.5	16.1 (0.0–92.7)	74.4	82.4 (5.1–100.0)	25.6	17.6 (0.0–94.9)	9.5	6.3 (0.0–100.0)
13	2.1	0.95 (0.0–53.4)	25.7	18.2 (0.0–95.8)	72.1	80.3 (2.8–100.0)	27.9	19.7 (0.0–97.2)	8.9	5.4 (0.0–89.7)
≥ 14	2.0	0.86 (0.0–24.9)	26.2	19.2 (0.0–93.1)	71.9	79.2 (5.2–100.0)	28.1	20.8 (0.0–94.8)	9.0	5.6 (0.0–100.0)
*P* value		0.54		**0.009**		**0.01**		**0.01**		**0.002**
*Current hormonal birth control use*										
No	2.6	1.18 (0.0–70.4)	27.3	19.9 (0.0–93.5)	70.1	77.8 (5.1–100.0)	29.9	22.2 (0.0–94.9)	9.6	6.5 (0.0–100.0)
Yes	2.7	1.48 (0.0–24.7)	29.2	21.2 (0.0–92.7)	68.2	76.4 (6.3–99.9)	27.0	23.6 (0.0–93.7)	9.7	6.8 (0.0–85.2)
*P* value		0.10		0.29		0.28		0.28		0.29
*Parity*										
Nulliparous	2.2	0.88 (0.0–25.1)	27.9	18.9 (0.0–93.5)	70.0	79.4 (5.1–100.0)	30.0	20.6 (0.0–94.9)	8.9	5.3 (0.0–100.0)
Parous	2.1	0.91 (0.0–70.4)	23.2	16.6 (0.0–95.8)	74.8	81.8 (2.8–100.0)	25.2	18.2 (0.0–97.2)	9.4	6.3 (0.0–100.0)
*P* value		0.44		0.13		0.06		0.06		** < 0.0001**
*Number of live births*										
0	1.9	0.74 (0.0–21.3)	26.8	21.2 (0.02–87.5)	71.3	76.7 (9.1–100.0)	28.7	23.3 (0.0–90.9)	7.6	5.3 (0.0–51.1)
1	1.8	0.91 (0.0–26.6)	23.4	16.8 (0.0–89.7)	74.8	81.8 (6.1–100.0)	25.2	18.2 (0.0–93.9)	8.9	5.8 (0.0–97.5)
2	2.1	0.98 (0.0–36.2)	22.7	15.6 (0.0–91.3)	75.2	82.5 (6.4–100.0)	24.8	17.5 (0.0 93.6)	9.8	6.7 (0.0–100.0)
≥ 3	2.1	0.87 (0.0–70.4)	21.1	15.0 (0.0–95.8)	76.9	83.6 (2.8–100.0)	23.1	16.4 (0.0–97.2)	10.1	6.9 (0.0–82.5)
*P* value		0.69		** < 0.0001**		**0.0003**		**0.0003**		** < 0.0001**
*Age at first full-term birth, years*										
< 25	2.0	0.83 (0.0–26.6)	21.6	15.0 (0.0–95.8)	76.4	84.1 (2.8–100.0)	23.6	15.9 (0.0–97.2)	8.7	6.3 (0.0–88.0)
25–29	2.1	1.06 (0.0–53.4)	23.4	18.7 (0.0–85.9)	74.4	79.9 (7.7–100.0)	25.6	20.1 (0.0–92.2)	8.9	5.9 (0.0–100.0)
≥ 30	2.1	1.08 (0.0–36.2)	23.4	17.2 (0.0–91.3)	74.5	81.2 (6.4–100.0)	25.5	18.8 (0.0–93.6)	9.1	5.7 (0.0–68.6)
*P* value		0.35		0.38		0.3612		0.36		**0.0038**
*Breastfeeding*										
Never	1.6	0.68 (0.0–70.4)	20.2	13.5 (0.0–95.8)	78.2	85.6 (2.8–100.0)	21.8	14.4 (0.0–97.2)	9.8	6.2 (0.0–97.5)
Ever	2.2	1.05 (0.0–53.4)	23.5	17.5 (0.0–91.3)	74.3	80.9 (6.1–100.0)	25.7	19.1 (0.0–93.9)	9.5	6.6 (0.0–100.0)
*P* value		**0.0002**		** < 0.0001**		** < 0.0001**		** < 0.0001**		**0.0002**
*Breastfeeding duration, months*										
Never	1.6	0.7 (0.0–70.4)	20.2	13.5 (0.0–95.8)	78.2	85.6 (2.8–100.0)	21.8	14.4 (0.0–97.2)	9.8	6.2 (0.0–97.5)
< 12	2.1	1.04 (0.0–26.6)	23.2	18.0 (0.0–91.3)	74.7	80.5 (6.1–100.0)	25.3	19.5 (0.0–93.9)	9.4	6.5 (0.0–100.0)
≥ 12– < 24	2.2	1.02 (0.0–36.2)	23.9	16.2 (0.0–89.3)	73.9	82.3 (8.6–100.0)	26.1	17.7 (0.0–91.4)	9.2	6.6 (0.0–65.9)
≥ 24	2.5	1.15 (0.0–53.4)	23.8	17.0 (0.0–90.5)	73.7	81.0 (7.7–100.0)	26.3	19.0 (0.0–92.2)	10.1	7.0 (0.0–91.0)
*P* value		**0.0016**		** < 0.0001**		** < 0.0001**		** < 0.0001**		**0.002**
*Bilateral oophorectomy*										
No	1.2	0.60 (0.0–18.4)	20.5	13.9 (0.0–90.6)	78.3	85.2 (9.2–100.0)	21.7	14.8 (0.0–90.8)	8.5	5.2 (0.0–93.7)
Yes	1.0	0.47 (0.0–12.0)	17.5	11.6 (0.0–79.3)	81.8	87.6 (20.4–100.0)	18.2	12.4 (0.0–79.6)	8.5	4.7 (0. 0–97.5)
*P* value		**0.01**		**0.02**		**0.02**		**0.02**		0.12
*Menopausal status*										
Premenopausal	2.6	1.26 (0.0–70.4)	28.1	20.6 (0.0–93.5)	69.3	77.1 (5.1–100.0)	30.7	22.9 (0.0–94.9)	9.7	6.6 (0.0–100.0)
Postmenopausal	1.2	0.56 (0.0–21.3)	19.1	12.6 (0.0–95.8)	79.7	86.2 (2.8–100.0)	20.3	13.8 (0.0–97.2)	8.4	5.0 (0.0–100.0)
Uterine Ablation	2.3	1.07 (0.0–18.4)	25.7	20.6 (0.0–89.9)	72.0	77.5 (7.8–80.5)	28.0	22.5 (0.0–92.1)	10.2	7.6 (0.12–74.0)
*P* value		** < 0.0001**		** < 0.0001**		** < 0.0001**		** < 0.0001**		** < 0.0001**
*Menopausal hormone therapy*										
Nonuser	1.2	0.53 (0.0–18.4)	18.6	12.7 (0.0–91.3)	80.2	86.2 (6.4–100.0)	19.8	13.8 (0.0–93.6)	8.8	5.2 (0.0–100.0)
Current user	0.8	0.45 (0.0 10.0)	20.3	12.2 (0.0–95.8)	78.9	87.2 (2.7–100.0)	21.1	12.8 (0.0–97.2)	6.2	3.1 (0.0–87.1)
Former user	1.4	0.52 (0.0–13.0)	19.9	15.3 (0.0–90.6)	78.7	83.5 (9.3–100.0)	21.3	16.5 (0.0–90.7)	8.0	5.3 (0.0–55.9)
*P* value		**0.0006**		0.63		0.67		0.67		** < 0.0001**
*Menopausal hormone therapy type-current user*										
Nonuser	1.2	0.53 (0.0–18.4)	18.6	12.6 (0.0–91.3)	80.3	86.2 (6.4–100.0)	19.7	13.8 (0.0–93.6)	8.8	5.1 (0.0–100.0)
Estrogen only	0.8	0.60 (0.0–5.2)	17.8	11.0 (0.0–71.6)	81.4	88.7 (25.1–100.0)	18.6	11.3 (0.0–74.9)	6.5	4.0 (0.0–87.1)
Combined estrogen and progesterone	0.8	0.32 (0.0–9.1)	22.9	14.7 (0.1–86.7)	76.3	85.1 (12.2–99.9)	23.7	14.9 (0.1–87.8)	6.3	2.5 (0.0–57.6)
*P* value		**0.04**		0.54		0.61		0.61		** < 0.0001**
*Menopausal hormone therapy type-former user*										
Nonuser	1.2	0.53 (0.0–18.4)	18.6	12.6 (0.0–91.3)	80.3	86.2 (6.4–100.0)	19.7	13.8 (0.0–93.6)	8.8	5.1 (0.0–100.0)
Estrogen only	1.2	0.60 (0.0–6.3)	19.4	15.8 (0.02–68.4)	79.5	83.3 (30.5–100.0)	20.5	16.7 (0.0–69.5)	8.0	5.5 (0.0–42.7)
Combined estrogen and progesterone	1.2	0.51 (0.0–6.2)	21.0	9.8 (0.0–90.6)	77.8	89.7 (9.3–100.0)	22.2	10.3 (0.0–90.7)	6.8	4.2 (0.0–55.9)
*P* value		0.68		0.76		0.77		0.77		0.33
*Family history of breast cancer*										
Negative	2.1	0.92 (0.0–53.4)	25.4	18.0 (0.0–95.8)	72.5	80.5 (2.8–100.0)	27.5	19.5 (0.0–97.2)	9.1	5.8 (0.0–100.0)
Positive	1.9	0.78 (0.0–70.4)	22.0	15.3 (0.0–93.3)	76.0	83.0 (5.3–100.0)	24.0	17.0 (0.0–94.7)	9.7	6. 2 (0.0–92.9)
*P* value		0.40		**0.002**		**0.003**		**0.003**		0.06

Asian/Other category comprised women who self-identified as Asian, Chinese, Filipino, Japanese, “Other” Asian, Asian/Native Hawaiian or Pacific Islander, etc. Parity: parous = women who had previously been pregnant, irrespective of the outcome; nulliparous = women who had never been pregnant. NLB = Number of Live Births (0 = previous pregnancies did not result in a live birth). *P* values were obtained using Kruskal–Wallis’s test. P values <0.05 were considered statistically significant and are indicated using bold fonts

**Table 3:** Beta coefficients and 95% confidence intervals (CIs) for the associations of host, reproductive, and lifestyle factors with quantitative tissue composition metrics among the 4108 women who donated normal breast tissue to the US-based Susan G Komen Tissue Bank that were included in the current study

Characteristic	Epithelium (%)		Stroma (%)		Adipose tissue (%)		Fibroglandular tissue (%)		Epithelium-to-stroma proportion (%)	
	*β* (95% CI)	*P* value	*β* (95% CI)	*P* value	*β* (95% CI)	*P* value	*β* (95% CI)	*P* value	*β* (95% CI)	*P* value
Age, years	− 0.013 (− 0.016; − 0.010)	** < 0.0001**	− 0.032(− 0.040; − 0.024)	** < 0.0001**	0.028 (0.022; 0.033)	** < 0.0001**	− 0.035 (− 0.043; − 0.027)	** < 0.0001**	− 0.001 (− 0.006; 0.004)	0.641
*Race/ethnicity*										
Non-Hispanic Caucasian	Ref (1.00)		Ref (1.00)		Ref (1.00)		Ref (1.00)		Ref (1.00)	
Black/African American	0.084 (0.013; 0.154)	**0.021**	0.038 (− 0.152; 0.227)	0.697	− 0.029 (− 0.161; 0.103)	0.670	0.066 (− 0.129; 0.261)	0.506	0.121 (− 0.005; 0.248)	0.060
Asian/Other	− 0.034 (− 0.127; 0.058)	0.469	− 0.152 (− 0.400; 0.095)	0.227	0.049 (− 0.124; 0.222)	0.581	− 0.152 (− 0.407; 0.103)	0.243	0.133 (− 0.032; 0.299)	0.115
*Education*										
High school/GED or less	Ref (1.00)		Ref (1.00)		Ref (1.00)		Ref (1.00)		Ref (1.00)	
Vocation/tech school or associates degree	− 0.024 (− 0.116; 0.067)	0.603	− 0.302 (− 0.547; − 0.058)	**0.015**	0.286 (0.115; 0.457)	**0.001**	− 0.306 (− 0.557; − 0.054)	**0.017**	− 0.030 (− 0.193; 0.133)	0.720
College degree	− 0.019 (− 0.101; 0.061)	0.629	− 0.169 (− 0.385; 0.047)	0.125	0.166 (0.015; 0.317)	**0.031**	− 0.175 (− 0.397; 0.048)	0.124	− 0.034 (− 0.178; 0.110)	0.643
Graduate/professional degree	0.021 (− 0.064; 0.108)	0.620	− 0.226 (− 0.456; 0.004)	0.054	0.212 (0.052; 0.373)	**0.010**	− 0.211 (− 0.447; 0.026)	0.080	0.057 (− 0.097; 0.210)	0.467
Other	− 0.038 (− 0.149; 0.074)	0.508	− 0.083 (− 0.381; 0.216)	0.587	0.129 (− 0.080; 0.337)	0.226	− 0.099 (− 0.406; 0.208)	0.528	− 0.140 (− 0.340; 0.059)	0.168
*Smoking*										
Never	Ref (1.00)		Ref (1.00)		Ref (1.00)		Ref (1.00)		Ref (1.00)	
Former	− 0.076 (− 0.143; − 0.008)	**0.028**	0.005 (− 0.176; 0.186)	0.954	− 0.029 (− 0.155; 0.098)	0.654	− 0.007 (− 0.193; 0.179)	0.958	− 0.069 (− 0.190; 0.052)	0.263
Current	− 0.035 (− 0.183; 0.113)	0.647	0.105 (− 0.291; 0.500)	0.605	− 0.111 (− 0.387; 0.165)	0.431	0.089 (− 0.318; 0.496)	0.665	0.047 (− 0.217; 0.311)	0.729
*Currently drink alcohol*										
No	Ref (1.00)									
Yes	− 0.030 (− 0.087; 0.028)	0.313	0.046 (− 0.108; 0.200)	0.555	− 0.036 (− 0.143; 0.072)	0.517	0.037 (− 0.121; 0.196)	0.645	− 0.080 (− 0.183; 0.023)	0.128
*Average number of alcoholic drinks per week*										
< 1	Ref (1.00)		Ref (1.00)		Ref (1.00)		Ref (1.00)		Ref (1.00)	
1–6	− 0.010 (− 0.119; 0.100)	0.863	0.288 (− 0.006; 0.581)	0.055	− 0.153 (− 0.358; 0.052)	0.145	0.281 (− 0.022; 0.583)	0.069	− 0.282 (− 0.478; − 0.086)	**0.005**
≥ 7	− 0.074 (− 0.223; 0.075)	0.329	0.249 (− 0.148; 0.646)	0.220	− 0.096 (− 0.374; 0.181)	0.495	0.214 (− 0.194; 0.623)	0.304	− 0.398 (− 0.663; − 0.133)	**0.003**
*Body mass index*										
< 25	Ref (1.00)		Ref (1.00)		Ref (1.00)		Ref (1.00)		Ref (1.00)	
25–29	− 0.126 (− 0.196; − 0.057)	** < 0.0001**	− 0.689 (− 0.873; − 0.503)	** < 0.0001**	0.508 (0.379; 0.638)	** < 0.0001**	− 0.702 (− 0.892; − 0.512)	** < 0.0001**	0.182 (0.059; 0.306)	**0.004**
≥ 30	− 0.171 (− 0.237; − 0.106)	** < 0.0001**	− 1.061 (− 1.234; − 0.888)	** < 0.0001**	0.773 (0.652; 0.894)	** < 0.0001**	− 1.074 (− 1.253; − 0.897)	** < 0.0001**	0.323 (0.208; 0.439)	** < 0.0001**
P trend		** < 0.0001**		** < 0.0001**		** < 0.0001**		** < 0.0001**		** < 0.0001**
*Age at menarche, years*										
≤ 12	Ref (1.00)		Ref (1.00)		Ref (1.00)		Ref (1.00)		Ref (1.00)	
13	0.024 (− 0.039; 0.086)	0.662	0.108 (− 0.058; 0.275)	0.202	− 0.120 (− 0.236; − 0.003)	**0.044**	0.113 (− 0.058; 0.284)	0.197	− 0.050 (− 0.161; 0.061)	0.379
≥ 14	− 0.015 (− 0.084; 0.055)	0.236	0.166 (− 0.018; 0.350)	0.077	− 0.110 (− 0.239; 0.018)	0.094	− 0.158 (0.032; 0.348)	0.102	− 0.063 (− 0.185; 0.060)	0.318
*P* trend		0.829		0.056		**0.044**		0.074		0.257
*Parity*										
Nulliparous	Ref (1.00)		Ref (1.00)		Ref (1.00)		Ref (1.00)		Ref (1.00)	
Parous	0.187 (0.125; 0.249)	** < 0.0001**	0.136 (− 0.030; 0.300)	0.108	− 0.062 (− 0.177; 0.054)	0.296	0.180 (0.009; 0.350)	**0.039**	0.187 (0.077; 0.298)	** < 0.0001**
*Number of live births*										
0	Ref (1.00)		Ref (1.00)		Ref (1.00)		Ref (1.00)		Ref (1.00)	
1	0.046 (− 0.117; 0.209)	0.578	− 0.390 (− 0.821; 0.040)	0.076	0.251 (− 0.039; 0.540)	0.090	− 0.359 (− 0.804; 0.086)	0.114	0.529 (0.239; 0.812)	** < 0.0001**
2	0.150 (− 0.008; 0.308)	0.064	− 0.488 (− 0.907; − 0.070)	**0.022**	0.250 (− 0.031; 0.532)	0.081	− 0.422 (− 0.854; 0.011)	0.056	0.753 (0.471; 1.035)	** < 0.0001**
≥ 3	0.157 (− 0.010; 0.324)	0.066	− 0.588 (− 1.030; − 0.147)	**0.009**	0.315 (0.017; 0.612)	**0.038**	− 0.509 (− 0.966; − 0.053)	**0.029**	0.826 (0.529; 1.123)	** < 0.0001**
P trend		**0.009**		**0.011**		0.082		**0.042**		** < 0.0001**
*Age at first full-term birth, years*										
< 25	Ref (1.00)		Ref (1.00)		Ref (1.00)		Ref (1.00)		Ref (1.00)	
25–29	− 0.043 (− 0.150; 0.065)	0.435	− 0.088 (− 0.371; 0.196)	0.544	0.049 (− 0.142; 0.240)	0.614	− 0.089 (− 0.382; 0.203)	0.550	0.043 (− 0.148; 0.233)	0.660
≥ 30	− 0.032 (− 0.150; 0.089)	0.591	− 0.109 (− 0.421; 0.202)	0.491	0.079 (− 0.130; 0.289)	0.457	− 0.108 (− 0.430; 0.213)	0.508	0.491 (− 0.089; 0.329)	0.260
*P* trend		0.703		0.874		0.708		0.847		0.440
*Breastfeeding*										
Never	Ref (1.00)		Ref (1.00)		Ref (1.00)		Ref (1.00)		Ref (1.00)	
Ever	0.060 (− 0.019; 0.139)	0.138	0.275 (0.065; 0.485)	**0.010**	− 0.151 (− 0.293; − 0.010)	**0.036**	0.276 (0.059; 0.493)	**0.013**	− 0.151 (− 0.292; − 0.010)	**0.036**
*Breastfeeding duration, months*										
Never	Ref (1.00)		Ref (1.00)		Ref (1.00)		Ref (1.00)		Ref (1.00)	
< 12	0.049 (− 0.037; 0.135)	0.260	0.266 (0.039; 0.492)	**0.022**	− 0.121 (− 0.273; 0.032)	0.120	0.263 (0.029; 0.497)	**0.028**	− 0.151 (− 0.303; 0.002)	0.052
≥ 12– < 24	0.051 (− 0.053; 0.154)	0.340	0.288 (0.014; 0.562)	**0.039**	− 0.182 (− 0.366; 0.003)	0.054	0.285 (0.002; 0.568)	**0.049**	− 0.188 (− 0.372; − 0.004)	**0.045**
≥ 24	0.0105 (− 0.012; 0.222)	0.076	0.305 (− 0.002; 0.613)	0.052	− 0.225 (0.432; − 0.018)	**0.033**	0.324 (0.006; 0.642)	**0.046**	− 0.136 (− 0.343; 0.071)	0.197
*P* trend		0.085		**0.035**		**0.015**		**0.033**		0.134
*Bilateral oophorectomy*										
No	Ref (1.00)		Ref (1.00)		Ref (1.00)		Ref (1.00)		Ref (1.00)	
Yes	− 0.103 (− 0.213; 0.007)	0.065	− 0.267 (− 0.600; 0.026)	0.074	0.216 (0.011; 0.420)	**0.039**	− 0.283 (− 0.585; 0.019)	0.066	− 0.109 (− 0.305; 0.087)	0.275
*Menopausal status*										
Premenopausal	Ref (1.00)		Ref (1.00)		Ref (1.00)		Ref (1.00)		Ref (1.00)	
Postmenopausal	− 0.158 (− 0.244; − 0.072)	** < 0.0001**	− 0.128 (− 0.357; 0.102)	0.276	0.035 (− 0.125; 0.195)	0.667	− 0.172 (− 0.408; 0.065)	0.154	− 0.280 (− 0.434; − 0.127)	** < 0.0001**
Uterine ablation	0.010 (− 0.156; 0.168)	0.938	− 0.001 (− 0.435; 0.432)	0.995	− 0.014 (− 0.316; 0.289)	0.930	0.001 (− 0.445; 0.447)	0.996	− 0.007 (− 0.297; 0.282)	0.961
*Family history of breast cancer*										
Negative	Ref (1.00)		Ref (1.00)		Ref (1.00)		Ref (1.00)		Ref (1.00)	
Positive	0.022 (− 0.044; 0.088)	0.510	− 0.153 (− 0.329; 0.023)	0.088	0.085 (− 0.038; 0.208)	0.175	− 0.138 (− 0.320; 0.043)	0.136	0.142 (0.024; 0.260)	**0.018**

Asian/Other category comprised women who self-identified as Asian, Chinese, Filipino, Japanese, “Other” Asian, Asian/Native Hawaiian or Pacific Islander, etc. Parity: parous = women who had previously been pregnant, irrespective of the outcome; nulliparous = women who had never been pregnant. Number of Live Births (0 = previous pregnancies did not result in a live birth). *β* coefficients and corresponding estimates were obtained using multivariable linear regression models that were mutually adjusted for epidemiological factors. Each tissue composition metric was square root transformed to approximate normality. The overall model included all women whereas breastfeeding, number of live births, and age at first full-term birth were modelled using information restricted to previously parous women. All statistical tests were 2-sided and *P* values < 0.05 were considered statistically significant and are indicated using bold fonts

**Fig. 2:**
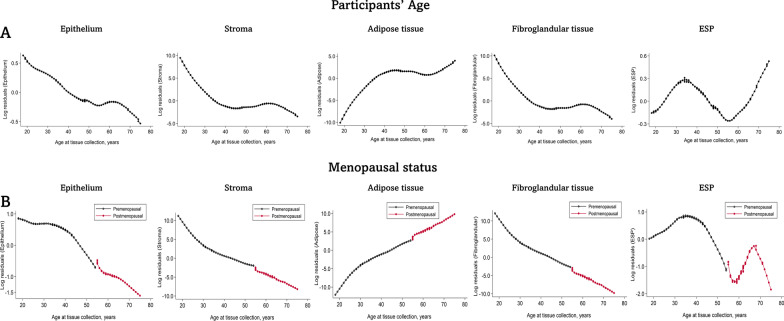
Relationship between age and menopause with quantitative tissue composition metrics of the normal breast. Locally weighted scatter plot smoothing (Lowess) functions were used to plot the residuals estimated in multivariable linear regression models for each tissue composition metric as a function of age. Lowess plots were constructed overall (**A**) and stratified by menopausal status (**B**). Pre- and postmenopausal status were defined by combining information on self-reported menopausal status, age (< 55 years (premenopausal) versus ≥ 55 years (postmenopausal)), bilateral oophorectomy, and having had a uterine ablation

Similar patterns of age- and menopause-related changes in tissue composition metrics as in the regression models were seen in Lowess curves for all the tissue composition metrics, with the exception of ESP. Unlike epithelium, stroma, and fibroglandular tissue that declined with increasing age, ESP showed a bimodal age distribution, increased starting at age 18 and peaked around 40 years of age, decreased from 40 until 55 years of age, and then increased with age thereafter (Fig. 2). Similar patterns of bimodal ESP distributions were seen with respect to menopausal status, with the first peak among premenopausal women occurring around age 30–40 years and a later peak among postmenopausal women occurring around 60–70 years of age. The bimodal age distribution of ESP corresponded to differences in the rates of decline of epithelial and stromal tissues by age. In multivariable linear regression models, stromal decline was ~ 34 times higher than epithelial decline before age 40 years but this slowed to ~ 2 times more between 40 and 55 years and increased again to ~ 10 times more after 55 years of age.

### Associations of reproductive factors with breast tissue composition metrics

Increasing age at menarche was associated with higher stromal and fibroglandular tissue but with lower adipose tissue and lower ESP (Table 2). Compared with women who had never been pregnant, previously pregnant women had statistically significantly higher ESP and this increased with increasing number of live births. Among parous women, those who breastfed had higher epithelium, stroma, fibroglandular tissue, and ESP but lower adipose tissue. In addition, increasing duration of breastfeeding was associated with increasing epithelium, stroma, fibroglandular tissue, and ESP but with decreasing adipose tissue (Table 2). The distributions of tissue composition metrics did not differ by age at first full-term birth.

In multivariable linear regression models (Table 3), parity and increasing number of live births remained statistically significantly associated with higher epithelium and higher ESP, with a strong linear trend in the magnitude of these associations with increasing stroma (Additional file 2: Fig. S2). Breastfeeding remained statistically significantly associated with increasing stromal and fibroglandular tissue and with decreasing adipose tissue and ESP (Table 3). Increasing duration of breastfeeding was associated with increasing stroma and fibroglandular tissue and with decreasing adipose tissue. Although different strata of breastfeeding duration were associated with ESP, there was no statistically significant trend in the association between duration of breastfeeding and ESP. The observed associations of parity, increasing number of live births, and breastfeeding with the individual tissue composition metrics were evident in both pre- (Additional file 5: Table S4) and post- (Additional file 5: Table S5) menopausal women.

**Fig. 3:**
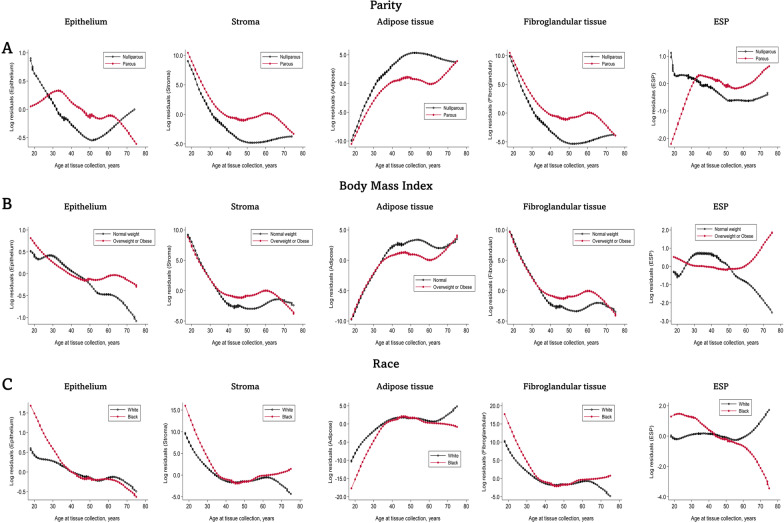
Relationships between parity, body mass index, and race with quantitative tissue composition metrics of the normal breast. Locally weighted scatter plot smoothing (Lowess) functions were used to plot the residuals estimated in multivariable linear regression models for each tissue composition metric as a function of age. Lowess plots were constructed separately for parous and nulliparous women, normal and overweight/obese women, and for Black and White women. Lowess plots were restricted to comparisons between Black and White women due to the small number of individuals in the other ethnic classes

Separate Lowess plots for nulliparous and parous women revealed rapid increase in ESP among parous women from 20 years, peaking around 40 years, declining slightly between 40 and 60 years, increasing again after 60 years, and remaining higher for parous than nulliparous women throughout life. Among nulliparous women, epithelium decreased progressively up to around 50 years after which it began to increase, surpassing levels in parous women around 65 years. In contrast, ESP decreased progressively up to 50 years among nulliparous women and remained fairly constant afterward (Fig. 3A).

### Associations of body mass index (BMI) with breast tissue composition metrics

The distributions of all tissue composition metrics varied by BMI categories (Table 2). Increasing BMI was associated with lower proportions of epithelium, stroma, and fibroglandular tissue but with higher proportions of adipose tissue (Table 2). Further, compared with normal weight women, overweight and obese women had statistically significantly higher levels of ESP. These associations persisted in multivariable linear regression models adjusted for other factors (Table 3). In separate plots for normal versus overweight/obese women (Fig. 3B), epithelial, stromal, and fibroglandular tissue components declined with age, while adipose tissue increased, in both groups. However, while decreases in epithelium (i.e., consistent with lobular involution) continued throughout life among normal weight women, age-related decline in epithelial tissue was not evident among overweight/obese women after 50 years. We also found ESP levels to be slightly higher among normal than overweight/obese women before 50 years of age, with a rapid decline among normal weight women after age 50 (Fig. 3B). Conversely, ESP levels were fairly constant among overweight/obese women before 50 years of age after which a rapid increase was observed causing ESP to be markedly higher among overweight/obese than normal weight women after 50 years (Fig. 3B).

### Associations of race and ethnicity with breast tissue composition metrics

Of the tissue composition metrics, the distributions of stroma and ESP varied statistically significantly by race and ethnicity (Table 2). In general, White women had the highest amount of stromal tissue (median = 18.0%), while Black women had the lowest (median = 15.1%). Conversely, ESP was highest among Black women (median = 6.7%) and lowest among White women (median = 5.7%). The difference in stroma by race/ethnicity was not statistically significant in multivariable models; however, epithelium was statistically significantly higher, while ESP was suggestively higher, among Black than White women (Table 3). In separate Lowess plots for Black and White women, the pattern of age-related decline in ESP differed by race. ESP was higher for Black than White women between 20 and 45 years of age, similar for Black and White women 40–60 years of age, and higher among White than Black women above 60 years of age (Fig. 3C). This pattern of association did not differ by parity status or BMI (Additional file 3: Fig. S3).

### Associations of other factors with breast tissue composition metrics

The distributions of individual tissue composition metrics varied according to several other factors, including FHBC, average number of alcoholic drinks per week, bilateral oophorectomy (Tables 2 and 3), as well as MHT use among postmenopausal women (Additional file 5: Table S5). While a positive FHBC was positively associated with ESP (Table 3), increasing number of alcoholic drinks per week (Table 3) and current use of combined estrogen and progesterone MHT formulation (Additional file 5: Table S5) were statistically significantly inversely associated with ESP in multivariable models. We did not find statistically significant associations between use of hormonal birth control among premenopausal women and any tissue composition metric.

## Discussion

By examining breast cancer risk factors in relation to quantitative tissue composition metrics of the normal breast, we showed that joint variations in both epithelial and stromal tissue composition may be critical for breast carcinogenesis. In particular, our findings suggest that both epithelial and stromal tissues involute toward fat and that imbalance in the rate of stromal and epithelial involution can manifest as high ESP, which may represent a feature of the mammary tissue ecosystem that is conducive for carcinogenesis [6]. The bimodal age- and menopause-related peaks in ESP that we found corresponds to the widely reported early-onset (premenopausal) and late-onset (postmenopausal) peaks in breast cancer incidence [20–22]. For most solid cancers, incidence rates increase linearly with age, but the pattern is different for female breast cancer which is characterized by an initial linear increase up to around age 50 years after which the slope changes to a downward trend and then resumes at a slower rate of increase with advancing age [22]. The point at which the incidence curve changes is known as the “Clemmensen’s” hook [23–25], a characteristic of female breast cancers that occurs around the perimenopausal period of life when ovarian function begins to decline until after its cessation at menopause.

Results from epidemiological studies have shown that the bimodal pattern of breast cancer incidence correlates with differences in breast tumor biology [22, 26]. Tumors occurring among younger/premenopausal women tend to be more aggressive than those occurring among older/postmenopausal women. However, tissue correlates of this phenomenon have yet to be fully characterized. Our findings of age- and menopausal-related bimodal ESP distributions suggest that epithelial and stromal tissues in the breast jointly respond to aging- and menopause-related changes in endogenous hormones. Anomalies at critical points in this process will manifest as variations in ESP that mirror and might explain the breast cancer incidence curve, Clemmensen’s hook, as well as age- and menopause-related differences in tumor biology.

Age-specific heterogeneity in breast cancer incidence and molecular subtype has been shown to characterize breast cancer risk relationships for parity, BMI, and race/ethnicity [27]. For instance, parity is associated with decreased breast cancer risk among older women but with increased risk among women younger than 30–44 years [27–30]. In the current study, parity was strongly associated with higher ESP, with statistically significant dose-dependent ESP increases with increasing number of live births. Observed associations between parity/increasing number of live births and ESP were driven by positive associations with epithelium and inverse associations with stroma, findings that are consistent with those from previous studies [10, 11]. We did not observe qualitative age interactions between parity and ESP. Instead, our observed bimodal age distribution of ESP was present among parous but not nulliparous women. The first ESP peak among parous women occurred around 30–45 years, which corresponds to the well-documented parity-related increased risk of early-onset breast cancer [31, 32]. The second ESP peak occurred after 55 years of age, and although not consistent with the documented protective effect of parity among older women [33], the apparent inconsistency may be due to etiologic heterogeneity of breast cancer with respect to parity/nulliparity [34, 35]. In general, parity is associated with increased risk of basal-like breast cancer, while nulliparity is associated with an increased risk of luminal breast cancer [32, 34, 36]. Although basal-like tumors tend to predominate among younger women, recent data have shown a bimodal age distribution in the incidence of this tumor subtype [37], which is consistent with our observation of a bimodal age distribution of ESP among parous women. In contrast to basal-like tumors, luminal tumors tend to predominate among nulliparous women and at older ages [38–42], which is also consistent with our findings of increasing epithelial tissue among older nulliparous as opposed to parous women.

Presumably, nulliparity might increase the risk of luminal breast cancer through an intrinsically epithelial-proliferation pathway while parity may increase the risk of basal-like breast cancer via stromal-epithelial crosstalk. The former idea is supported by results from studies showing strong associations between nulliparity and highly proliferating luminal tumors, defined by expression of the proliferation marker Ki67 [43] and is buttressed by data from experimental studies showing that parity induces terminal differentiation of luminal epithelial cells as well as downregulation of growth factors and the upregulation of growth inhibitory signals [44]. Conversely, parity may increase risk of aggressive/basal-like breast cancers by disrupting stromal-epithelial homeostasis, a notion that is supported by studies showing that stromal remodeling and perturbed immune response mechanisms constitute pathways by which parity influences breast cancer risk [3, 45–47]. In addition to the strong association that we observed between parity and increasing ESP, our observations that the magnitude of this association increased with increasing stromal (as opposed to adipose tissue) content support the potential role of stromal-epithelial crosstalk in mediating parity-related breast carcinogenesis. In the current study, breastfeeding was associated with lower ESP but not epithelium. A previous study reported an inverse association between breastfeeding and adipose tissue content, which is consistent with our findings [11]. Breastfeeding is thought to attenuate parity-related increased risk of aggressive breast cancers [48, 49]. Conceivably, our finding of an inverse association between breastfeeding and ESP, which appears to be driven by increased stromal content with increasing breastfeeding duration, may suggest that breastfeeding’s protective effect might be partly mediated through post-lactational stromal restoration.

The association of elevated BMI with breast cancer incidence varies by age [27, 50]. Among women younger than 50 years, being overweight or obese is associated with decreased breast cancer risk, but risk increases among these women thereafter. In the current study, we found a strong association between elevated BMI and increasing ESP. Differences in ESP between women with normal versus overweight/obese BMI were highest after 50 years of age, corresponding to the age period during which elevated BMI is associated with increased breast cancer risk. Among women younger than 50 years, however, overweight/obese BMI was associated with slightly lower ESP than normal BMI, which is consistent with the lower risk of breast cancer among women with overweight/obese than normal BMI below 50 years of age [51]. The relatively higher ESP among normal than overweight/obese women between 30 and 50 years appears to be due to the correspondingly lower stromal proportion among women with normal BMI. On the other hand, the markedly higher ESP among overweight/obese than normal weight women after 50 years of age appears to be driven by a combination of increasing epithelium and decreasing stroma. These tissue-level observations reflect the complex relationships between BMI, aging, and breast cancer risk among pre- and postmenopausal women [52].

Our findings might also hold clues into differences in age-related incidence and tumor biology among racial groups [53]. We found that ESP was higher among Black than White women before 40 years, but this declined with advancing age in parallel with increasing ESP among White women leading to a crossover around 55 years, after which ESP levels were higher among White than Black women. It is unclear why ESP levels were higher among younger Black than White women and vice versa among older women, but this pattern is reminiscent of the higher rates of early-onset breast cancer among Black than White women and of later-onset breast cancer among White than Black women [53]. Although this analysis was based on self-reported race and ethnicity, our findings are consistent with those from a previous analysis within this population that found TDLU levels to be higher among women of African than European genetic ancestry [54]. Given the link between higher TDLU levels and TNBC [55, 56], our findings with respect to epithelial and ESP differences by race buttress the notion that changes in mammary tissue composition may reflect cumulative exposure to endogenous and exogenous breast cancer risk factors over the lifespan, holding clues into the etiopathogenesis of breast cancer subtypes.

Having a positive FHBC is a strong risk factor for breast cancer development. However, the tissue pathways by which FHBC influences breast cancer risk are yet to be fully defined. Results from a previous study suggested that polygenic risk scores for breast cancer development were associated with TDLU involution [57]. Here, we found positive FHBC to be associated with higher ESP, which is consistent with its association with increased breast cancer risk. We also found varying but less consistent associations between other risk factors and individual tissue composition metrics. Having had a bilateral oophorectomy was suggestively associated with lower epithelial and fibroglandular tissue, a low-risk tissue phenotype that is consistent with the reduced risk of breast cancer among women who have had a bilateral oophorectomy [58, 59]. Current use of MHT, particularly the combined estrogen and progesterone formulation, was associated with higher stromal and fibroglandular tissue, correspondingly lower adipose tissue, and lower ESP. While the association between MHT use and higher fibroglandular tissue is consistent with its association with higher mammographic density [60], a radiological representation of the amount of fibroglandular tissue in the breast, and elevated breast cancer risk [61, 62], its association with lower ESP is not consistent with its risk increasing role. Similar to MHT use, we found increasing number of alcoholic drinks per week to be inversely associated with ESP. In line with data from epidemiological studies suggesting that use of combined MHT and alcohol consumption are associated with elevated risk of hormone receptor-positive (ER + , mostly low grade) but not receptor-negative (ER-, mostly high grade) breast cancers [43, 63–69], our observed associations of combined MHT use and alcohol consumption with breast tissue composition metrics may provide further clues into the role of variations in exposure-tissue interactions in the etiopathogenesis of breast cancer subtypes.

This study has several important strengths, including the application of high-accuracy machine learning algorithms for the detailed and centralized assessment of quantitative tissue composition metrics on digitized, H&E-stained, biopsy specimens from over 4000 normal breast tissue donors. To the best of our knowledge, this is the largest analysis of its kind to date to investigate associations between several questionnaire-based risk factors and quantitative tissue composition metrics of the normal breast. The large sample size allowed us to conduct analysis overall and stratified by menopausal status and other relevant characteristics. We were able to control for several potential confounders in our analyses and to conduct relevant sensitivity analyses, all of which strongly support the internal validity of our findings. Nevertheless, the current analysis is not without limitations. For instance, we were unable to examine longitudinal changes in tissue composition metrics. Also, we were unable to directly evaluate the potential impact of sociodemographic, environmental, and socioeconomic factors on our BMI and race-related findings, but all our estimates were adjusted for educational level as a surrogate for socioeconomic status. The use of questionnaire-based data may be associated with measurement error and recall bias. However, the accuracy and reproducibility of self-reports for many of the factors that were significantly associated with breast tissue composition metrics in the current study have been previously documented to be high [70–72]. Moreover, measurement errors in exposure assessment are very unlikely to be differential by tissue composition metrics and, if they exist, will be more likely to bias the results toward the null. We did not have information on time since last pregnancy or time since weaning, so we were unable to evaluate temporal changes in the magnitude of the associations between pregnancy or weaning and tissue composition metrics. Nevertheless, pregnancy and breastfeeding history were significantly associated with ESP even in advanced ages suggesting that time since last pregnancy or breastfeeding may not confound our observed associations. Although this study was based on a population of self-selected volunteers, BCRAT (or Gail) scores of absolute breast cancer risk for participants in this study were normally distributed (Additional file 4: Fig. S4), as in the general population, which lends credence to the external generalizability of the findings. Nevertheless, the majority of the study participants were US-based, otherwise healthy, White women, which might impact the generalizability of these findings to other racial groups or populations.

In conclusion, we investigated the relationships of host, lifestyle, and reproductive factors on quantitative tissue composition metrics of the normal breast, including epithelium, stroma, adipose tissue, fibroglandular tissue, and histologic ESP (a metric of the proportion of fibroglandular tissue that is epithelium relative to stroma). We found several established breast cancer risk factors to be associated with individual tissue metrics, including novel observations with respect to ESP. In particular, age, menopausal status, parity, breastfeeding history, BMI, race, FHBC, alcohol intake, and MHT use demonstrated heterogenous associations with ESP consistent with their documented associations with incidence of molecular breast cancer subtypes. Overall, our findings provide critical insights into the role of stromal-epithelial interactions in breast cancer etiology, with implications for our understanding of the histogenesis of breast cancer subtypes. Conceivably, variations in tissue composition metrics on biopsy, particularly ESP, could serve as intermediate markers of risk and might be used to inform breast cancer prevention strategies for women.

### Supplementary Information

**Additional file 1. Fig. S1**: Flow diagram showing the exclusion and exclusion criteria employed in this analysis.

**Additional file 2. Fig. S2**: Magnitude of the associations between parity and epithelial composition of the normal breast according to the amount of stroma on the slides.

**Additional file 3. Fig. S3**: Associations between race and epithelium-to-stroma proportion (ESP) by parity and body mass index (BMI).

**Additional file 4. Fig. S4**: Distribution of BCRAT (or Gail) scores of absolute breast cancer risk for participants in this study.

**Additional file 5. Table S1**: Frequencies of missing values for each variable included the current analysis of 4108 women from the Komen Tissue Bank Study. **Table S2**: Associations of host, reproductive, and lifestyle factors with epithelium-to-stroma proportion among healthy women volunteers participating in the Komen Tissue Bank project, using missing values indicators, and following multiple imputation (N = 4108). **Table S3**: Associations of risk factors with epithelium-to-stroma proportion among healthy women volunteers participating in the Komen Tissue Bank project, with and without adjustment for age at first full-term birth (AFFB). **Table S4**: Associations of host, reproductive, and lifestyle factors with quantitative tissue composition metrics among healthy premenopausal women participating in the Komen Tissue Bank project (N = 2696). **Table S5**: Associations of host, reproductive, and lifestyle factors with quantitative tissue composition metrics among healthy postmenopausal women participating in the Komen Tissue Bank project (N = 1412).

## Data Availability

The images and questionnaire-based data analyzed during the current study are available from the Komen Tissue Bank and can be viewed, downloaded, or requested on their website: https://virtualtissuebank.iu.edu/. Data on tissue composition metrics analyzed in the current study can be obtained from the corresponding author on reasonable request.
