# Supplementary figures and images for: Host, reproductive, and lifestyle factors in relation to quantitative histologic metrics of the normal breast
Source: Breast Cancer Res. 2023 Aug 15;25:97. doi: 10.1186/s13058-023-01692-7 (PMC10426057; doi:10.1186/s13058-023-01692-7)

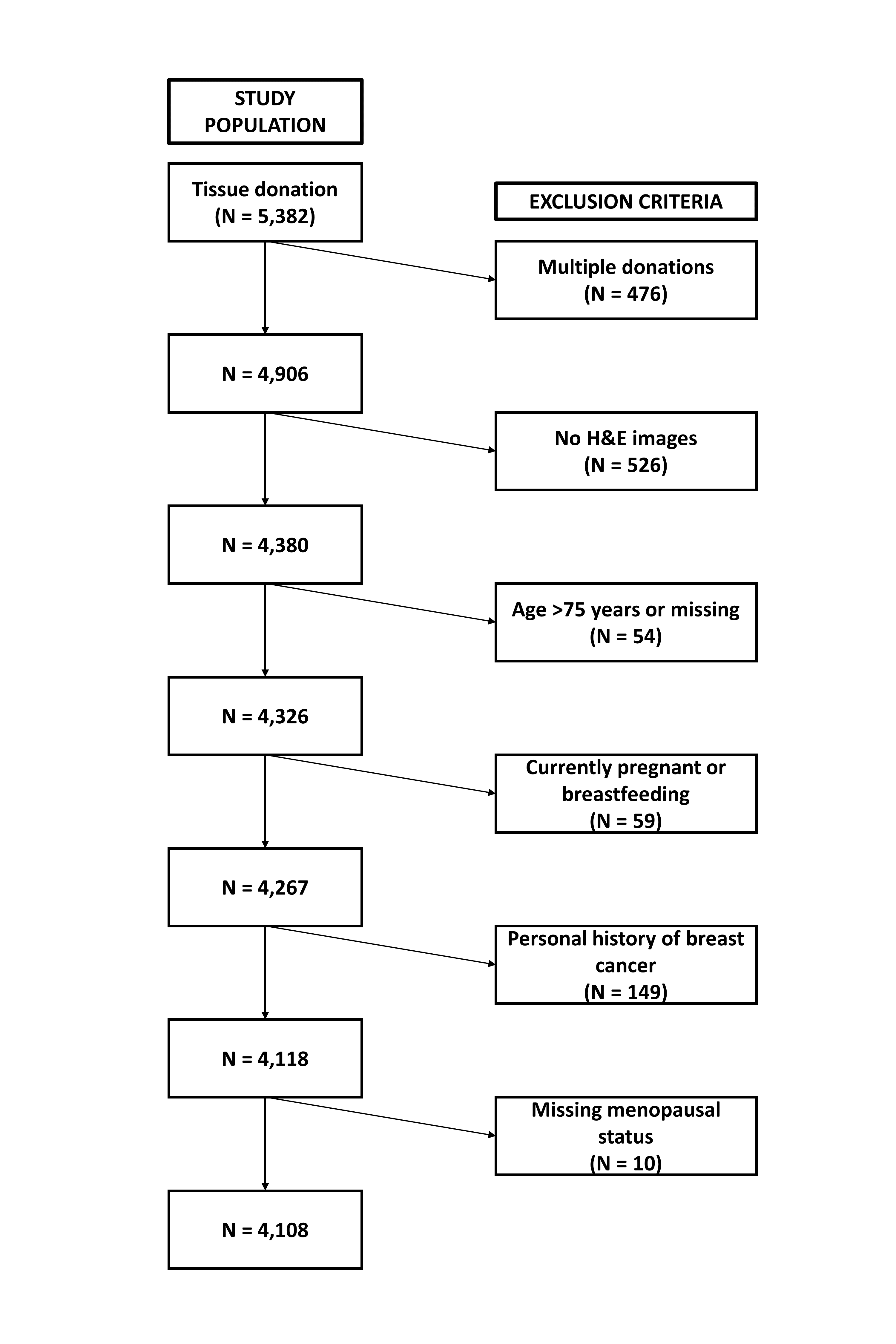

Supplement: Supplementary file 1 — Additional file 1. Fig. S1: Flow diagram showing the exclusion and exclusion criteria employed in this analysis. [file 13058_2023_1692_MOESM1_ESM.png]

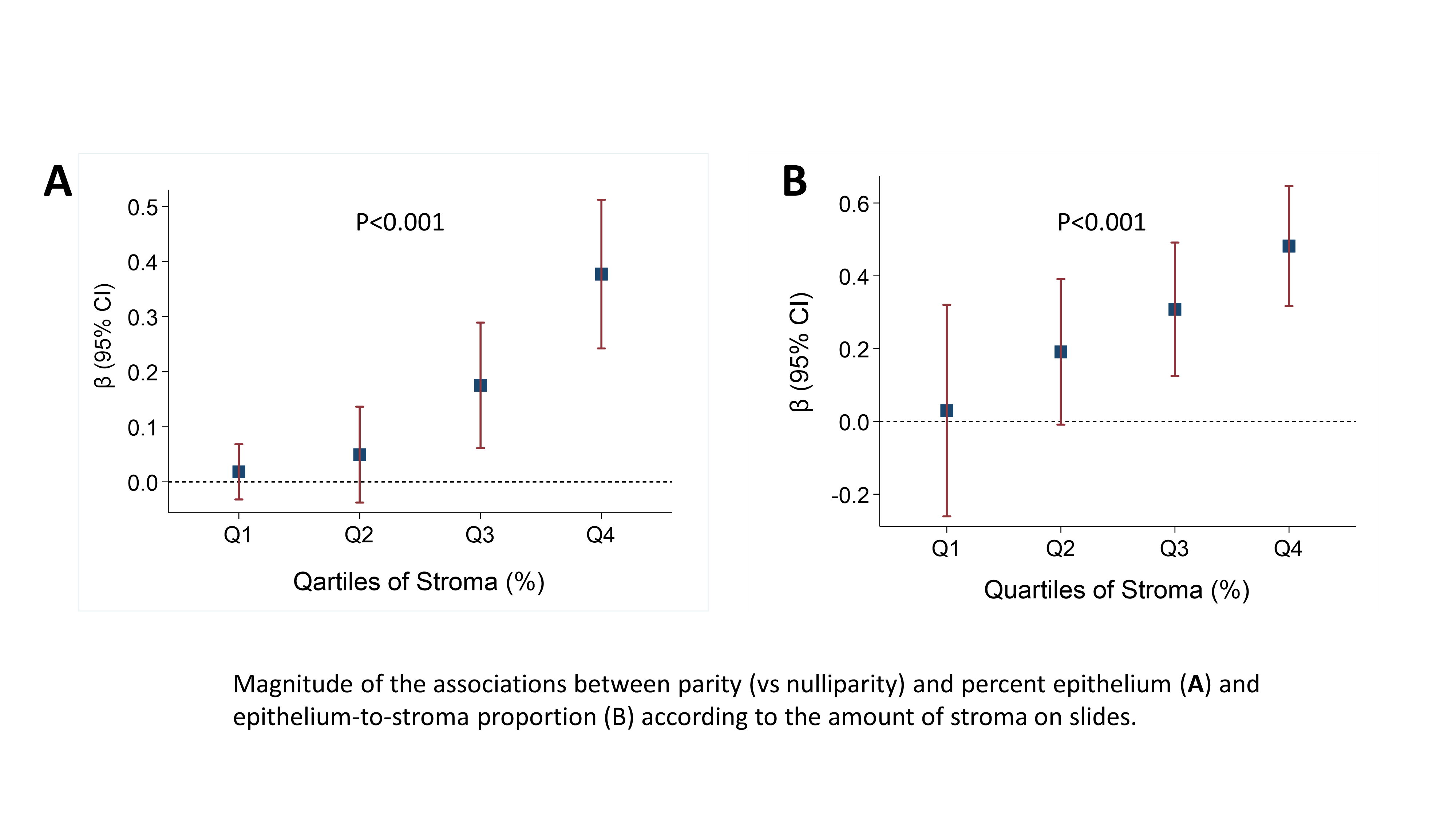

Supplement: Supplementary file 2 — Additional file 2. Fig. S2: Magnitude of the associations between parity and epithelial composition of the normal breast according to the amount of stroma on the slides. [file 13058_2023_1692_MOESM2_ESM.tif]

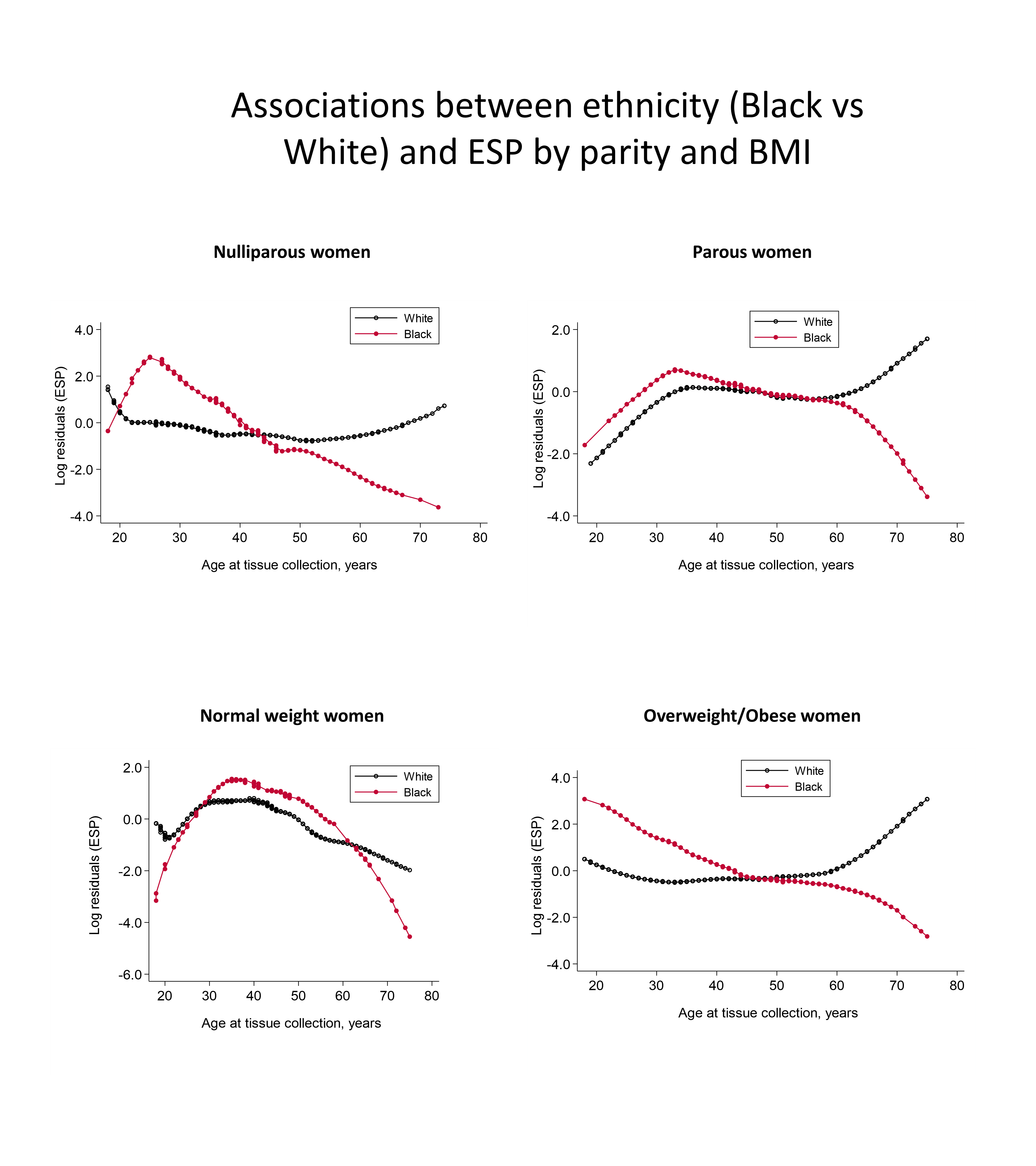

Supplement: Supplementary file 3 — Additional file 3. Fig. S3: Associations between race and epithelium-to-stroma proportion (ESP) by parity and body mass index (BMI). [file 13058_2023_1692_MOESM3_ESM.png]

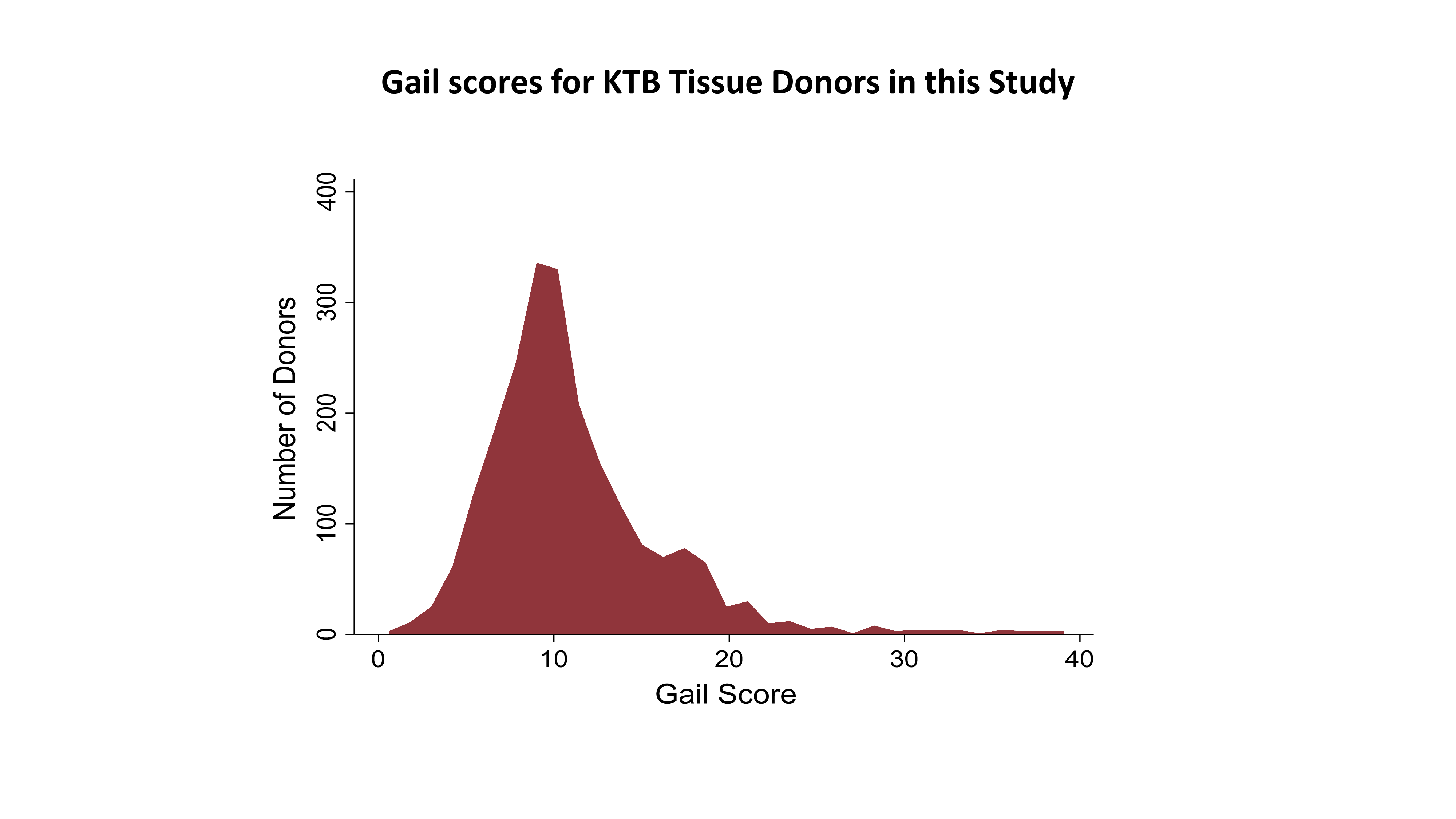

Supplement: Supplementary file 4 — Additional file 4. Fig. S4: Distribution of BCRAT (or Gail) scores of absolute breast cancer risk for participants in this study. [file 13058_2023_1692_MOESM4_ESM.tif]
